# Synthesis and Characterization of Hydrogenated Diamond-Like Carbon (HDLC) Nanocomposite Films with Metal (Ag, Cu) Nanoparticles

**DOI:** 10.3390/ma13071753

**Published:** 2020-04-09

**Authors:** Loukas Koutsokeras, Marios Constantinou, Petros Nikolaou, Georgios Constantinides, Pantelis Kelires

**Affiliations:** Research Unit for Nanostructured Materials Systems (RUNMS), Department of Mechanical Engineering and Materials science and Engineering, Cyprus University of Technology, Limassol 3046, Cyprus

**Keywords:** hydrogenated diamond like carbon, nanocomposite thin films, silver nanoparticles, copper nanoparticles, cluster beam source

## Abstract

In this work, the synthesis and characterization of hydrogenated diamond-like carbon (HDLC) nanocomposite thin films with embedded metallic Ag and Cu nanoparticles (NPs) are studied. These nanocomposite films were deposited using a hybrid technique with independent control over the carbon and metal sources. The metallic nanoparticles were directly deposited from the gas phase, avoiding surface diffusion of metal species on the deposition surface. The structural features, surface topography and optical properties of pure and nanocomposite HDLC films are studied and the effect of metal introduction into the carbon matrix is discussed. The interactions between the carbon ion beam and the NPs are considered and it is demonstrated that the nanocomposite HDLC:metal films, especially for Cu NPs, can retain the transparency level of the pure HDLC, by limiting the interactions between metal and carbon during deposition.

## 1. Introduction

Amorphous carbon (a–C) and its hydrogenated form (a–C:H) have attracted significant attention in the scientific community due to their interesting properties such as chemical inertness, low friction coefficient, wear resistance, optical transparency, and biocompatibility, among others [1,2,3,4,5,6]. All these properties arise from the complexity and the configurational possibilities of the amorphous network pertinent to C–C and C–H bonds. The fraction of the sp^3^ and sp^2^ sites as well as the hydrogen content are the most influential characteristics of the material that subsequently dictate its properties. Amorphous carbon with high sp^3^/sp^2^ bonds-ratio is called Diamond Like Carbon (DLC) and has properties resembling crystalline diamond [7]. The hydrogenated form of DLC is commonly abbreviated as HDLC and its properties are affected also by the hydrogen content [8,9]; it is a material with many photonic applications [10,11]. The deposition of DLC and HDLC films is usually based on some variant of the chemical vapor deposition (CVD) technique, such as capacitively coupled plasma enhanced CVD (PECVD), which is the most commonly used deposition method, electron cyclotron resonance CVD (ECR CVD) and microwave plasma CVD (MPCVD), among others [4,12,13]. Additionally, physical vapor deposition (PVD) methods have been used to deposit DLC and HDLC films, such as magnetron sputtering (MS), high power impulse magnetron sputtering (HiPIMS) [14,15] and pulsed laser deposition (PLD) [16].

The addition of metal nanoparticles in an amorphous carbon matrix (hydrogenated or not) can lead to multifunctional materials with properties such as high catalytic activity [17], enhanced solar absorption [18,19] and nanoscratch resistance [20,21,22,23], among others. For photonic and plasmonic applications especially, a transparent HDLC matrix is desired in order to support the localized surface plasmon resonance (LSPR) of metallic NPs, such as Au, Ag and Cu. The intensity and position of the LSPR peak are affected by the NPs size, distribution, shape, interparticle distance and also by the refractive index of the matrix.

The techniques used to produce HDLC nanocomposites are usually realized by the deposition of metal species by sputtering in a reactive hydrocarbon atmosphere [14,24], or by metal ion implantation and aggregation/growth via post growth annealing [25]. The reactive sputtering approach, which is widely used, deposits nanocomposite thin films with a broad range of metal content that ranges from 0 to 60% atomic. The size of the NPs is usually in the range of 2 to 25nm and their average size is also coupled with the metal content [26,27]. Furthermore, the distance between particles is small especially for high metal concentration. Until now the LSPR peak has been observed only for metal contents above 3% for Ag and 9% for Cu NPs in similar HDLC nanocomposites [26,27,28].

The absence of the LSPR manifestation in lower metal concentrations we believe is due to the reduction of the HDLC transparency. The introduction of metals in the HDLC matrix induces graphitization [21,28,29,30], which in turn reduces the optical transparency of the films due to π-π* transition [11] (of the sp^2^ network). For low metal contents, the concentration of plasmonic NPs is not enough to overcome the loss of transparency. High transparency levels of the HDLC matrix are required for LSPR manifestation at lower metal contents.

In this work, we study the optical properties of HDLC nanocomposites with Ag and Cu NPs focused on their potential for plasmonic and photonic applications such as antireflective coatings and sensors. A novel CVD/PVD hybrid deposition system is used where matrix and metal nanoparticles are deposited from different sources. The HDLC matrix is deposited by an RF ion beam using a CH_4_/Ar plasma and the NPs by a nanocluster beam source. For comparison we deposited thin films of pure HDLC, plain NPs and a combination of both (nanocomposites) and we discuss the effect of the metals to the transparency of the HDLC matrix and the relative LSPR manifestation. The HDLC matrix features are discussed after the addition of metals by comparing the effect of the π-π* transition and the plasmon resonance in the optical spectrum.

## 2. Materials and Methods

All samples were deposited on Czochralski-grown commercial silicon (001) and quartz (c-cut) wafers and depositions were carried out in a high vacuum chamber with basic pressure lower than 10^−5^ Pa, pumped down by a turbomolecular and a dry scroll pump. All substrates were ultrasonically cleaned for 10 min in acetone and methanol baths and dried in nitrogen flow. An ion beam source (RFMAX60, Mantis Deposition Ltd., Thame, UK) with an RF inductively coupled plasma was used to produce the hydrogenated amorphous carbon films. The ion beam source consisted of a quartz cup surrounded by an RF coil and a set of two highly transparent graphite grids located at the exit of the cup. RF power (at 13.56 MHz) applied to the coil, ionized the gaseous species in the quartz cup and produced an inductively coupled plasma with high ionization degree. The plasma lost electrons at the walls of the quartz cup and obtained a positive plasma potential which was in the order of 40–50 V. Through the inner graphite grid, the plasma potential could be raised to a selected value and this created a voltage drop with the outer grounded grid. This voltage drop extracted and accelerated ions from the plasma to the substrate, forming a beam with narrow kinetic energy distribution of ions. The diameter of the beam was 2 inches with an incident angle of 30° from the substrate’s surface normal.

Metal NPs were deposited directly from gas phase by a NP source (Nanogen50, Mantis Deposition Ltd., Thame, UK). This source was a modified magnetron sputtering with a gas aggregation zone where nucleation and growth of the sputtered species occurred in the gas phase. The NPs were formed inside this aggregation zone where vapors from the sputtering target nucleated and grew due to high argon pressure and gas phase collisions. The NPs exited the aggregation zone through a narrow aperture of 5 mm diameter. The stream of NPs and gas atoms after exiting though the aperture, expanded into a cone (divergent beam) due to the pressure differential between the aggregation zone and the main chamber. In line with the NP source, a quadrupole mass spectrometer (MesoQ, Mantis Deposition Ltd., Thame, UK) was used to detect the mass distribution of the formed NPs and act as a filter with accuracy 0.2% in size. This hybrid deposition system was presented into more detail in a previous publication [21].

For this study, a varying methane to argon gas mixture ratio of 3:1, 2:1 and 1:1 was introduced into the quartz discharge chamber of the ion beam while the RF power was kept at 200 W and the inner grid voltage was set to 100 V. The working pressure of the chamber was between 0.02 and 0.05 Pa during all depositions and substrates were kept at ground potential, while the deposition time for all samples was 30 min. Silver and copper sputtering targets of 4 and 5 N purity, respectively, were used for the fabrication of NPs and Ar (purity 5 N) was used as the sputtering gas. The discharge current and voltages were 30 mA and 250 V and 65 mA and 260 V for the Ag and Cu targets, respectively.

The optical response of the films were measured by a UV/Vis spectrometer in reflectance mode. The complex refractive index coefficients (n, k) were extracted by fitting the experimental reflectivity measurements with an air/HDLC/Si three layer model in combination with a classical Lorentz oscillator dispersion relation. Fitting to the experimental data was performed using the RefFIT software (v1.3) [31].

X-ray reflectivity (XRR) and grazing incidence X-ray diffraction (GIXRD) measurements were performed in a diffractometer (Rigaku Ultima IV, Tokyo, Japan), equipped with a Cu tube operated in 40 kV and 40 mA. The X-ray beam was shaped by a curved multilayer mirror in parallel mode (Rigaku CBO optic, Tokyo, Japan) and by divergent and receiving slits without knife edge. The experimental XRR data were fitted using GlobalFit software (v2.0.4) from Rigaku.

The topographic characteristics for all films were collected with a Scanning Probe Microscope (Ntegra Prima, NT-MDT, Moscow, Russia) using intermittent contact mode atomic force microscopy (IC-AFM) with controlled humidity (~30%) and room temperature conditions. Images of 3 μm × 3 μm were collected by oscillating a flexible cantilever with a sharp probe (NSG10) upon the surface of the materials while keeping the scanning rate at 1Hz. The qualitative and quantitative characteristics of the images were evaluated using the open software package, Gwyddion [32].

## 3. Results and Discussion

### 3.1. Pure HDLC Films

**Figure 1:**
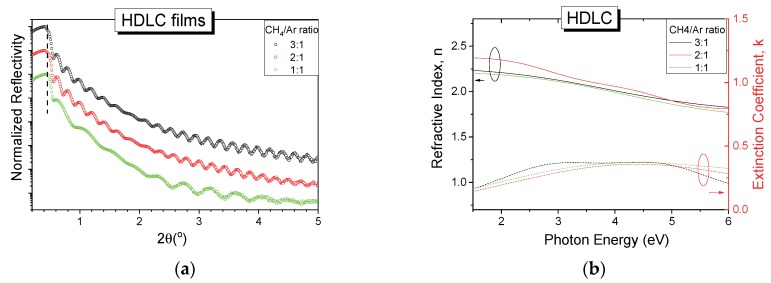
(**a**) Normalized X-ray reflectivity (XRR) patterns (with vertical shift for clarity) and (**b**) optical constants n, k for pure hydrogenated diamond-like carbon (HDLC) thin films deposited with different CH_4_/Ar gas ratio.

As a starting point in the effort to deposit HDLC:metal nanocomposites, pure hydrogenated amorphous carbon films, without any metal NPs, were grown. Three samples were deposited using CH_4_ to Ar ratio values (CH_4_/Ar) of 3:1, 2:1 and 1:1. We had chosen to vary the gas mixture as a mean to control the deposition rate of the thin films instead of RF power in consideration of the particular characteristics of the ion beam source. The normalized XRR patterns of the pure HDLC samples are shown in Figure 1a, with an offset in the vertical axis intentionally introduced for clarity.

All reflectivity curves have similar patterns and show fringes up to 5° angle which indicate high quality and smooth thin films. The critical angle of the curves, indicated with a vertical dashed line in Figure 1a, which relates to the mass density of the films, remains unaffected by the gas ratio used implying that the density of the films is approximately the same in all deposited films. The experimental reflectivity data were fitted in a three-layer model (film/SiC/Si) using GlobalFit software, and the density was found to be 2.0 g/cm^3^ for all films independently of the gas ratio used. The thicknesses of the films were found to be 40, 38 and 20 nm for the 3:1, 2:1 and 1:1 CH_4_/Ar ratio respectively. The amplitude changes of the reflectivity curves in the angular range between 1° and 2° 2θ are attributed to the formation of an intermediate layer between the silicon substrate and the amorphous hydrogenated carbon film due to the high kinetic energy of impacting species which is above the sub-plantation threshold [33,34,35]. The roughness of the thin films was obtained from the fitting process and was found to be between 0.3 and 0.5 nm for all samples.

The specular reflectance in the UV/Vis range of the deposited HDLC samples was measured and by appropriate fitting of the experimental curves using a two-layer model (film on Si) the optical constants of the film were obtained. Figure 1b shows the extracted n and k values for the three HDLC films grown with different gas ratios. All samples show similar behavior in this spectral range, further supporting the XRR results. The values of n range from 2.25 to 1.90 for the low and high energy photons respectively and the maximum value of k does not exceed 0.4. These values are in good agreement with similar samples grown using an ion beam [36]. The optical properties of hydrogenated amorphous carbon are governed by their bonding characteristics and the hybridization of the carbon atoms, and specifically the sp^3^/sp^2^ ratio and the composition of hydrogen. The sp^3^ fraction of our samples was estimated in a previous work [21] by Raman spectroscopy and it was in the order of 50%, while the hydrogen content was 20%–25%. The above results place the samples under the category of HDLC films.

### 3.2. HDLC with Metal NPs

**Figure 2:**
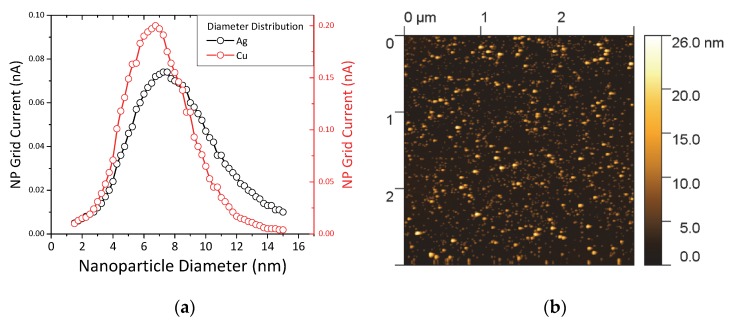
(**a**) Grid current intensity versus nanoparticle (NP) diameter for silver (black circles) and copper NPs (red circles). (**b**) Atomic force microscopy (AFM) image of pure Ag NPs on Si wafer.

The HDLC samples containing metal NPs were fabricated by co-deposition of carbon through the ion beam source and metallic clusters through the NP source. The experimental parameters for the ion beam were kept the same as the pure HDLC films while the NP source was configured to provide the maximum flux of NPs with the widest diameter range. The main deposition parameters that affect the diameter range of the NPs are (apart from the material) the length of the aggregation zone, the sputtering power and the gas flow. The configuration of the source was performed by monitoring the inline mass spectrometer (MesoQ filter) and by changing the aforementioned parameters we established the desired NP distribution. Figure 2a shows the normalized current intensity of the produced NPs versus the NP diameter for Ag (black circles) and Cu (red circles). The diameter of the NPs ranges between 1 and 15 nm for both materials, while the center of the peaks is between 7 and 8 nm. The inline filter allows for pre-selecting a single size diameter NPs with an accuracy of ±2% but in this study during deposition we allowed the full size spectrum of generated NPs to pass through, in order to achieve a higher deposition rate on the sample surface.

Figure 2b shows a 3 μm × 3 μm AFM image of pure Ag NPs deposited on Si wafer for 30 min. The NPs are distributed randomly and homogenously on the surface and the coverage is high but not complete. The brighter spots are pile-ups of two or three NPs considering their diameter distribution from the graphs of Figure 2a and their shape appears round as expected from the gas phase nucleation and growth which promotes spherical shapes. In a previous work we verified that the size detected by the inline filter matches very well with the actual size of the NPs by a combined AFM and High-Resolution Transmission Electron Microscope (HRTEM) characterization [21]. One question that we expect to resolve in this study is how these NPs would interact with the high kinetic energy carbon/argon atoms arriving from the ion beam source.

**Figure 3:**
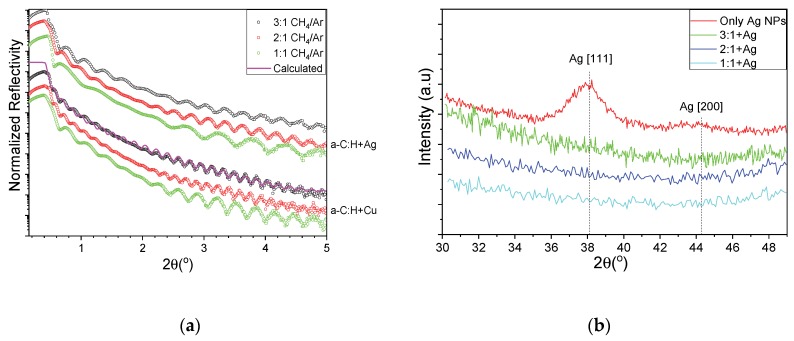
(**a**) XRR curves for HDLC containing Ag (top group) and Cu (bottom group) and (**b**) grazing incidence X-ray diffraction (GIXRD) patterns of pure NP film (red) and HDLC nanocomposites of varying CH_4_/Ar ratio.

It is known that the filling factor/metal content of the nanocomposites with metal inclusions plays a significant role in the final properties of the films [18]. In this study, we have changed the CH_4_/Ar ratio in order to vary the deposition rate of the carbon matrix while keeping the metal NP flux constant. Figure 3a shows the XRR experimental curves of HDLC thin films containing Ag (top group) and Cu (bottom group). The experimental curves were fitted to a three-layer model (film/interface layer/substrate) and a representative calculation is shown with a magenta line overlaying the corresponding experimental curve in Figure 3a. From the fitting, the density of the samples is found to be 2 g/cm^3^ for all samples, independently from the containing metal. The incorporation of the metal nanoclusters did not alter the measured density of the films which might be explained by either the low metal content and the low graphitization of the films [18,29]. The thicknesses of the samples were 28 to 16 nm for the AgNPs nanocomposites and 49 to 28 nm of the Cu-NPs nanocomposites. From the shape of the curves one can see that no layering has been detected in the bulk of the thin films which implies a homogeneous distribution of the metallic NPs along the growth direction. The root mean square roughness of all samples with Ag and Cu, as estimated through XRR measurements, was between 0.4 and 0.6 nm. Figure 3b shows the GIXRD patterns of pure NP film (red) and HDLC nanocomposites of varying CH_4_/Ar ratio. Only the sample with freestanding Ag NPs exhibits a clear Ag [111] peak and a weak [200] peak. The [111] peak also appears symmetric and very close to the angular position of a bulk sample (noted with vertical dashed lines), implying a soft-landing regime and a stress-free lattice constant. The nanocomposite samples do not exhibit any diffraction peaks, suggesting a rather small number of NPs per unit area and thus a low metal concentration.

**Figure 4:**
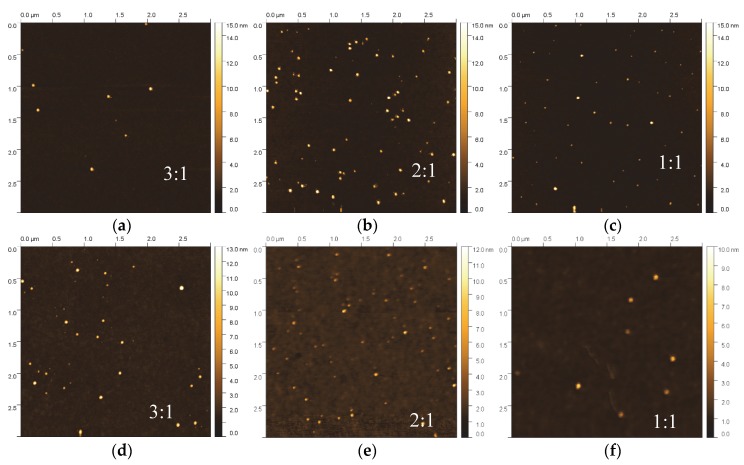
(**a**) AFM 3 μm × 3 μm scans for HDLC/Ag (**a**–**c**) and (**b**) HDLC/Cu (**d**–**f**) samples grown at various CH_4_/Ar ratios (3:1, 2:1 and 1:1).

Figure 4 shows a grid of AFM scans of the composite thin film samples grown with different CH_4_/Ar ratio and the same NP flux as well as size distribution, as shown in Figure 2a. Clearly, for all samples the metal NPs are observed in the scans without any signs of aggregation or coalescence, however their surface coverage compared to the sample without HDLC decreased. The root mean square roughness (*R*_q_) lies within the range of 0.2 and 0.8 nm for all samples, while the average roughness (*R*_a_) exhibits lower values in the range between 0.08 and 0.27 nm. Both definitions of roughness reside in the sub-nanometer regime and relate primarily to the surface characteristics of the amorphous carbon matrix deposited within this study. The contribution of the metallic NPs plays an insignificant role due to the very low concentrations. Additionally, the root mean square values of roughness obtained through AFM (0.2–0.8 nm) are in close agreement with the values obtained through XRR analyses (0.3–0.6 nm). The roughness definition more heavily affected by the presence of NPs is the peak to valley height (*R*_z_) which shifts in the tenths of nanometers regime (13.7 to 16.7 nm for HDLC:Ag and 17.3 to 17.7 nm for HDLC:Cu) consistent with the size of NPs grown within this study and unaffected by their quantity or content. It is interesting to note that the type of metal or the CH_4_/Ar ratio does not influence *R*_z_ which is consistent with the fact that the size of the NPs is independently controlled through the deposition setup/mass filter.

The reduction in the number of NPs can be explained by the energetic conditions during ion beam deposition of HDLC. The incoming carbon/argon ions are partially re-sputtering NPs already deposited at the sample surface, creating in some cases surface voids that are subsequently filled with amorphous carbon. This explanation can be strengthened by the sporadic craters appearing in the flat background, mostly for the Cu-containing samples. For this reason, to deposit nanocomposites with higher NP concentration, the re-sputtering should be taken into consideration and the deposition process should be adapted accordingly. This can be circumvented either by increasing the deposition flux of the NPs to compensate for their losses or by using an alternative approach such as a sequential deposition of the matrix and the NPs.

Assessing the observed reduction in the number of NPs per unit area from the AFM scans (Figure 4) it appears that the metal loading is rather low, estimated in the order of sub 1%. Even at this low metal content, the properties of the nanocomposite differ from the properties of the pure matrix. Low metal NP content has been found to enhance the nanoscratch resistance and partially relieve the residual stress [21] of the matrix and induce antimicrobial properties [37]. In similar nanocomposite systems and also as shown by ab initio calculations, a reduction in the sp^3^ content was observed [18,29] due to graphitization of the HDLC matrix around the NPs. The graphitization of the matrix should increase the optical absorbance of the films, which might or might not be desirable, depending on the application. For photonic and plasmonic applications a reduction in the transparency of the matrix is not desirable and needs to be avoided.

**Figure 5:**
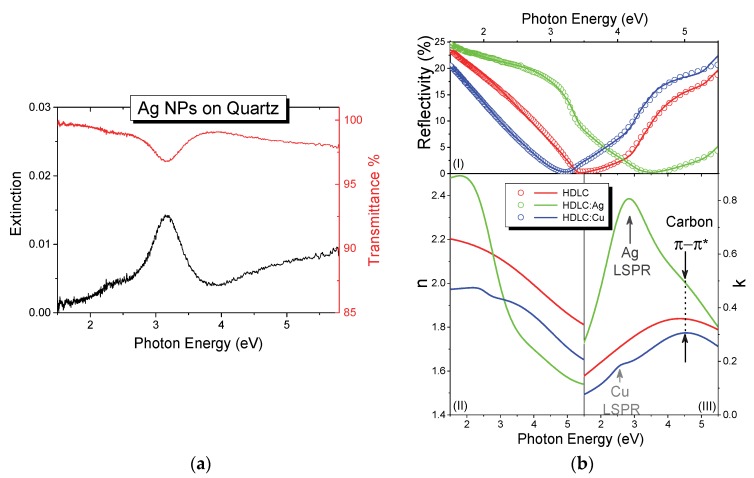
(**a**) Extinction (left, black) and transmittance (right, red) spectra of pure Ag NPs deposited on quartz. (**b**)(top) Normalized reflectivity (open circles) and their corresponding fit curves; the extracted (bottom left) n and (bottom right) k values of the pure HDLC (black), HDLC:Ag (green) and HDLC:Cu (blue) nanocomposites.

The transmittance and extinction spectra of the pure Ag NPs sample deposited on quartz are shown in Figure 5a. A distinct absorption peak is observed at 3.16 eV, which is due to LSPR of the metallic Ag NPs. The absorption value is well correlated with the LSPR absorption shown by similar sizes of Ag NPs [38]. This resonance originates from the collective oscillation of the free electrons of the NPs, and affected by the size of the NPs, the distance between them and the optical properties of the surround matrix [39]. The top of Figure 5b shows the measured experimental reflectivity of the HDLC; HDLC:Ag and HDLC:Cu thin films for 2:1 CH_4_/Ar ratio and their corresponding fit lines for each curve. All curves show a drop in the spectra between 3 and 4.5 eV, which is due to the evolution of a reflectivity fringe due to the transparency and the thickness of each sample; thicker samples would exhibit multiple reflectivity fringes in this range. The overlaid lines demonstrate the calculated reflectivities and all three curves show very good agreement to the experimental data.

The bottom part of Figure 5b shows the extracted n (left) and k (right) values of the thin films as obtained from the fitting process. The values of n and k for the HDLC and HDLC:Cu samples are close to each other indicating similar optical behavior before and after the metal doping. The values of n are in the range between 1.65 and 2.2 eV for the high and low energy part of the spectrum. The highest value of the extinction coefficient k, in the measured range is located around 4.5 eV, which is very similar to the pure HDLC value. Additionally, a faint peak was observed at 2.6 eV which is assigned to the LSPR of Cu NPs. The refractive index of nanocomposites with Ag (Figure 5b, bottom left) has even larger value span from 1.45 to 2.45 in comparison to pure HDLC. The addition of Ag NPs resulted in higher absorption and a broad peak in the spectrum, around 2.7 eV. The same peak is also observed in the 3:1 CH_4_/Ar ratio grown sample but not in the 1:1, possibly due to low film thickness and the poor fitting convergence of the latter. This peak is assigned to the LSPR of Ag as [14,40,41], which is more pronounced in comparison with HDLC:Cu as expected.

The manifestation of both LSPR peaks for Ag and Cu NPs indicates that the HDLC matrix retains its transparency after the metal addition, as also shown by the comparison between the pure HDLC. Table 1 shows the position of the π-π* transition of the matrix and the LSPR as extracted from the fitting process. The spectral position of the π-π* transition is coupled with the bonding characteristics of the carbon matrix and it shifts to lower energies for smaller sp^3^/sp^2^ ratio. By comparing the values of Table 1, we observe a reduction in the energy of the π-π* transition from 5.08 to 4.65 and 4.85 eV for Ag and Cu respectively. This redshift indicates a graphitization of the matrix which on the other hand is reduced, compared with other metal containing carbon nanocomposites [42]. The values of the second oscillator are indicating the position of the LSPR absorption which are also in accordance with similar nanocomposite system [43,44].

## 4. Conclusions

We have demonstrated the successful deposition of pure HDLC, pure metal NPs and HDLC:metal nanocomposites with embedded Ag and Cu NPs through the utilization of a novel CVD/PVD hybrid deposition system in which the formation of NPs is decoupled from the HDLC matrix deposition via the novel deposition system design. The pure HDLC samples exhibit transparency and the values of refractive index were between 2.25 to 1.90 for the low and high energy photons respectively. The density was found to be 2.0 g/cm^3^ and the surface roughness was in the sub-nanometer scale. AFM scans show that the nanocomposite HDLC thin films contained spherical, non-agglomerated NPs of Ag or Cu, with controlled size distribution between 1 and 15 nm, verifying the output of the quadrupole mass spectrometer located in line with the cluster beam source. A reduction in the number of NPs was noted between pure NPs films and HDLC:metal nanocomposites due to the resputtering of surface and poorly attached NPs by the ion beam during the co-deposition. The UV/Vis measurements demonstrated that the addition of metal NPs, especially for Cu in the HDLC films resulted in retaining the optical transparency and response of the nanocomposite HDLC films, at the same level as the pure HDLC. This was achieved by reducing the interactions of the metal and the matrix and keeping the graphitization of the amorphous carbon matrix network low. The nanocomposite thin films with Ag NPs exhibit a similar level of absorbance with the pure HDLC films, with an additional broad peak located at 2.7 eV which is assigned to the manifestation of the LSPR of Ag NPs. The average roughness of the nanocomposite thin films for both metals was low, again in the sub-nanometer scale. These results demonstrate that the decoupling of the NPs formation from the HDLC matrix can reduce the graphitization of the matrix, enabling the development of such types of nanocomposite that can be exploited in various applications ranging from protective coatings to photonic devices.

## Figures and Tables

**Table 1:** Oscillator positions for pure and metal containing HDLC.

Sample	Oscillator Position for π-π* (eV)	Oscillator Position for LSPR (eV)
HDLC	5.08	-
HDLC:Ag	4.65	2.75
HDLC:Cu	4.85	2.57
